# Bayesian analysis on missing visual information and object complexity on visual search for object orientation and object identity

**DOI:** 10.3758/s13414-024-02901-x

**Published:** 2024-05-22

**Authors:** Rachel T. T. Nguyen, Matthew S. Peterson

**Affiliations:** 1 Human Factors and Applied Cognition, Psychology Department, George Mason University, David King Hall, Room 2086, 4400 University Drive, MSN 3F5, Fairfax, VA 22030-4422 USA; 2 Psychology Department, Cognitive Behavioral Neuroscience, George Mason University, Fairfax, VA USA; 3 Psychology Department, Neuroscience Interdisciplinary Program, George Mason University, Fairfax, VA USA; 4 Center for Advancing Human-Machine Partnerships, Psychology Department, George Mason University, Fairfax, VA USA

**Keywords:** Occlusion, Visual search, Attention, Amodal completion

## Abstract

Missing visual information, such as a gap between an object or an occluded view, has been shown to disrupt visual search and make amodal completion inefficient. Previous research, using simple black bars as stimuli, failed to show a pop-out effect (flat search slope across increasing visual set sizes) during a feature search when the target was partially occluded, but not in cases where it was fully visible. We wanted to see if this lack of a pop-out effect during feature (orientation) search extended to complex objects (Experiment 1) and identity search (Experiment 2). Participants completed orientation and identity visual search tasks by deciding whether the target was present or not present. Bayesian analyses was conducted to find evidence for observed data to be under the null (pop-out effects) or alternative hypotheses (differences in search slopes). When no occluders or gaps were present, a pop-out effect occurred when searching for a simple objects' orientation or identity. In addition, object complexity affected identity search, with anecdotal evidence suggesting that some complex object may not show a pop-out effect. Furthermore, white occluding bars were more disruptive than having a gap of visual information for feature search but not for identity search. Overall, pop-out effects do occur for simple objects, but when the task is more difficult, search for real-world objects is greatly affected by any type of visual disruption.

## Introduction

When we search for an everyday object, such as a coffee mug, it is helpful to rely on one or more features to guide attention. By using top-down processing, we can use a defining feature (such as orientation or color) to search for a target amongst distractors. For example, in a kitchen cupboard, mugs can be similar in color, such as white with different logos. If there is a stemmed wine glass you enjoy using, you could search through rows of stemless drinking glasses serially until you find your target. However, this method is inefficient and unnatural when there is a better solution. Instead, if you search in parallel for the glass’s distinguishing feature (e.g., having a stem or glass foot to hold up the glass cup), this search method will efficiently guide your attention to your favorite stemmed wine glass, without wasting time examining all of the drinking cups and glasses. However, during your search through the kitchen cabinet, some objects will be partially hidden or occluded; these object arrangements can affect our search behavior, and this behavior may depend on which objects and how these objects occlude other objects.

### How partially occluded objects are processed attentively

#### Amodal completion

**Fig. 1:**
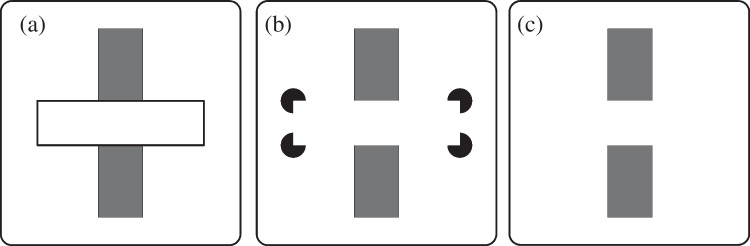
Amodal, modal, and effortful object completion (with gap). There is a gray bar in each image, with a partial lack of visual information. By amodal completion (**a**), you can visualize a complete gray vertical bar behind the horizontal white bar. In (**b**), an illusory object leads to *modal* completion: a complete horizontal white bar overlayed on top of four circles behind the white rectangle’s corners. In contrast, when there is a gap between two gray vertical segments (**c**), there are no object boundaries that make it easy to visualize the gray bar segments extending across the gap to complete a perceptual whole object, and instead we interpret it as two separate vertical gray bars

Our visual system works to make sense of what we can see, even if some visual information is missing or not detected. We can identify common whole objects, and we also understand that they do not always appear entirely visible when viewed. When parts of objects are hidden, we do not perceive those parts as non-existent pieces: instead, we perceive partially occluded objects as a whole – not as objects defined by the sum of its visible parts (an aspect of Gestalt theories) (Michotte et al., 1991, as cited by Wertheimer, 1994). When an object is partially occluded, we can imagine the piece that is occluded, a phenomenon labeled called amodal completion processing (Kanizsa, 1979, as cited by Rensink & Enns, 1998). This phenomenon involves early perceptual processing of an incomplete object to give us the perception of a complete object (Nanay, 2018). In Fig. 1, although we can interpret the first image (a) as two short vertical gray bars and a horizontal white bar, instead, we naturally see it as one long gray bar that extends “behind” a white bar.

#### Modal and effortful object completion

##### Modal completion

In contrast to amodal competition, there are two other forms of object completion involving occlusion that we will be discussing: modal completion and effortful object completion. *Modal* completion occurs when an occluder's edges blend in with the background, yet the occluded object appears to be “beneath” the occluder (Singh, 2004). In Fig. 1, in the second image (b) a white horizontal bar is visible, even though there are no connecting outlines because four black circles can be seen “beneath” this white bar (through amodal completion). Comparing instances where amodal and modal completion occurs, this gap of information is caused by an object that is obstructing the view and at least one object that is obstructed from view.

##### Effortful completion

In contrast to modal completion, gap conditions do not have any contours that imply a vivid figure camouflaged against its background. Instead of easily perceiving the third image (c) containing one long vertical gray bar, it is easier to visualize two short vertical gray bars (Fig. 1). Indeed, the law of prägnanz would predict that two vertical bars is the simplest solution. Looking at the image, the object does not automatically appear to be one cohesive unit, but rather, object pieces that belong to two objects (see Fig. 1c). By top-down perceptual processing, one can determine that the two individual vertical pieces could be part of the same object – and this process is effortful. And while there are these differences between amodal, modal, and effortful object completions, the one thing they all have in common is a visual disruption of information that makes search more difficult compared to when objects are fully visible (Davis & Driver, 1998; Rensink & Enns, 1998; Wolfe et al., 2011).

### Does amodal completion occur in search for real-world objects?

The question we want to ask is: Does amodal completion occur in visual search for both simple and real-world objects? From past research examples, we know that this perceptual filling is possible for simple objects from experiments involving basic stimuli, such as bars (Wolfe et al., 2011), lines (Rensink & Enns, 1998), and letters such as “T” and “L” (Treisman & Gormican, 1988). Visual search tasks commonly involve searching for the presence of a feature, such as the search for the presence of an object orientation. If an object is partially occluded, amodal completion may be necessary for feature searches. For simple objects, this perceptual filling is a process that can occur automatically or preattentively (He & Nakayama, 1992; Rensink & Enns, 1998). Amodal completion could possibly occur automatically for common everyday objects as well, but that does not mean that search behavior for partially occluded simple shapes predicts search behavior for partially occluded complex everyday objects. Amodal completion might not occur automatically in search for everyday objects. After all, people, cars, and man-made objects are more visually complex than alphabet letters or monolithic solid bars. Though simple objects can increase in complexity with added contiguous shapes and segments, for the sake of this paper, simple objects refer to stimuli such as bars, lines, and letters and complex shapes to real-world objects. What differs between our definition of increased object complexity and those of traditional lab-created complexities is that familiar real-world object holds both semantic and episodic memories associated with the stimuli that may affect behavior; these additional properties are not always apparent in complex geometric shapes that are occasionally included in studies.

To know how well findings from past research are applicable to search for real-world objects, it is necessary to compare search behaviors for fully visible objects and partially occluded objects at increasing object complexity (initially, from simple objects like rectangles to real-world objects that are slightly more complex like rectangular remote controllers). This comparison in search behaviors for objects of different complexity allows us to better understand how visible parts of objects or partially occluded objects are processed.

### Understanding preattentive grouping effects between an object’s visible parts

We can better understand how these types of objects are processed by understanding conditions that lead to object pieces being grouped preattentively. By examining search behaviors when searching Müller-Lyer type figures, Rensink and Enns (1995, 1998) found that object segments are preattentively processed as a group when: (1) object sections (e.g., line segments from an object) line up directly across a gap, or (2) line up directly against an object occluder. Some of the experiments are described below to show how these findings apply to the types of “simple” stimuli. It is not yet known if attention is deployed the same way (e.g., preattentive grouping of object parts) for complex objects (which we define as real-world objects).

#### Active grouping effects across an occluding object and gap

##### Occluding object

When an object appears to have another object on top of it or in between its segments, grouping effects for object segments occur preattentively when the segments line up directly across from one another. In a series of experiments, Rensink and Enns asked participants to find and determine whether the target’s overall length is the same or different from the distractors (1995) or to find and determine whether the length of a target’s segments is the same or different from the distractors' segment lengths (1995, 1998). Search times for the segment difference task were slower than the overall length task, indicating that the segments were grouped and not readily accessible individually.

**Fig. 2:**
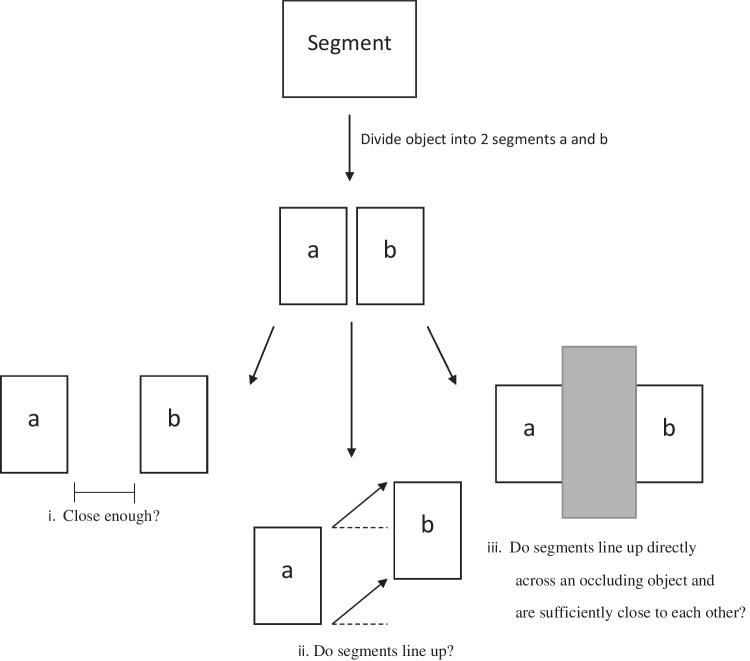
Preattentive grouping effects determined by object segment placement conditions. Findings from Rensink and Enns (1995, 1998). (i) If there is a gap of visible information between object segments, preattentive segment grouping occurs at sufficiently short distances. Segments line up across a gap. (ii) Segments grouped preattentively if segment edges line up across from each other. This example shows edges that do not line up, and pre-emptive grouping does not occur when object segments are displaced across a gap and do not line up. (iii) Segments are grouped preattentively if an object is placed between segments that line up across from each other

Objects can be defined as a physical composition of its individual parts (or object “members”), but there are times when perceptual grouping occurs between parts or components at early stages of vision such that the individual components are not readily accessible during search (Rensink & Enns, 1995). Because this perceptual grouping can occur at early vision and occurs automatically without effort, search for the entire object can be easy, yet it becomes difficult to access or decipher an object’s individual parts/line segments. Overall, some level of grouping effect occurs pre-emptively (at early vision) when object segments touch the occluder and are evenly displaced across it (see Fig. 2).

##### Gap

They also found that when there is a visible gap between line segments, search differed depending on how an object’s segments line up with each other across a gap of information (see Fig. 2). The segment pieces must sufficiently close in distance and line up across from one another. If the line’s segments orient in a way that the segments could extend and connect with each other (within a sufficiently short distance), the gap of information is pre-emptively processed and a rapid grouping effect between object segments occurs (“assembly-based search”). If the line segments were slightly displaced in the same or a different direction, having a gap in the middle of the line segment changes the search task to a “junction-based search,” where there is no automatic grouping between segmented parts. In summary, minute differences in gap length between the target (solid line) does not increase search difficulty (because grouping effects can occur at lower-level vision), but when a gap slightly displaces the segmented parts of an object, an object’s segmented parts are not grouped in early vision, allowing the individual segments to be readily accessible.

#### Do automatic grouping effects occur across gaps of information for complex objects?

This automatic grouping of object segments when there is a sufficiently small gap of information in the middle (or lack of pre-emptive grouping with displaced object parts) might not occur for more complex objects. It is also possible that these automatic grouping effects may occur for some objects with varying object complexity.

### Amodal completion process in visual search

While Rensink and Enns (1998) found longer search times for targets in most conditions that had an occluder compared to a diagonal gap that created mismatched object pieces (in comparison with objects cleanly intersected with a gap), Wolfe and colleagues (2011) examined whether amodal completion could make search for partially occluded objects as efficient as fully visible bars. To their surprise, they found that it did not. Using orientation as a feature to distinguish targets from distractors, they found occluder/gap-specific effects between search slopes for finding the stimuli (e.g., black bar). In their Experiment 1, black bars were either fully visible, occluded by a white bar placed diagonally on top, fully visible and placed above a white diagonal bar, or had a gap in the middle of the black bar so that it looked like two mirrored black triangles separated by space sharing the same gray color as the background. One point to make is that a gap in the middle of the stimuli removed the same visual information as the white occluding bars. The task was to determine if a vertical black bar was present among distractors composed of horizontal black bars. When occluders were absent, search slopes were flat, indicating that the vertical bar “popped out” and was easy to find. They found steeper search slopes across increasing visual set sizes for targets that were partially occluded by white bars compared to when there was a gray gap or when there were no occluders (Wolfe et al., 2011). In addition, search was less efficient when gray gaps were present compared to the fully visible condition. Overall, when simple objects are visually intersected with a lack of visual information (gap and occluder conditions), searching for the whole object’s orientation was difficult. This task became even more difficult when the lack of visual information was due to an occluding object.

One possibility for these results is that Wolfe and colleagues’ tasks used simple black bars as their stimuli, and amodal completion may not be occurring pre-emptively. If it did occur pre-emptively, then the black bar’s orientation (or “verticalness”) should exist for the completed figure and allow it to “pop out.” Alternatively, amodal completion only occurs when the object is attended, negating any chance for pop-out search to occur. In the gap and white occluder conditions, if the black bar pieces grouped pre-emptively, the target orientation should “pop out” and be easily found; there would not be a significant difference in search slopes between the no occluder and the white occluder conditions. Even though the black bar pieces line up directly across from each other in the gap and white occluder conditions (a configuration where Müller-Lyer segments grouped preattentively in Rensink and Enns (1995) experiments), search slopes were steeper than search slopes for whole black bars. Since this feature search stresses being able to distinguish the outline from the correct groups of triangles, the task may add some difficulty in correctly grouping the correct triangles together. In the occluder condition, when searching for the black vertical bar, attention may first deploy to the white occluding bar (because it appears closer to the viewer) before the black bar; this attentional deployment may induce a difference in search slopes between the gap and white occluder conditions. Nonetheless, these results would suggest that amodal completion does not occur preattentively.

Another possibility for these results is that amodal completion does not occur when targets are defined by a single feature, such as vertical, and instead might only occur when searching for a whole object (Pratt & Sekular, 2001). This lack of an occurrence might be due to the level of processing at which attention is allocated. For example, when searching for a simple feature, such as orientation, there is increased activation in early areas of visual processing, such as V4 (Maunsell & Treue, 2006; Zhou & Desimone, 2011). In contrast, object-based attentional effects occur at higher levels, namely the Lateral-Occipital Complex (Hou & Liu, 2012). This difference in localization in the stream of processing is important, because the final amodally completed representations appear to be represented in the Lateral-Occipital Complex (Weigelt et al., 2007). Taken together, these results suggest that a visual-search task that emphasizes object-based, rather than feature-based, representations may be more likely to benefit from amodal completion.

### How is amodal completion affected in searches for real-world objects?

We used a series of visual search tasks to test whether amodal completion is also observed for complex, real-world objects. Of importance is our Experiment 2, in which the task emphasized searching for the target's identity rather than an incidental feature such as orientation. Specifically, features such as orientation can be considered a transient property, rather than part of an object’s identity. For example, a shovel is still considered a shovel, whether it is leaning against a toolshed or laying on the ground. In addition, we do not know if grouping effects for object pieces occur preattentively across different levels of object complexity when the object is partially occluded or missing. Previously described experiments included solid lines and bars as their stimuli. By using real-world objects as our stimuli, we can either (1) find that past research with simplistic stimuli predicts search behavior for real-world objects, or (2) find that attention is deployed differently in search for partially visible real-world objects.

### Defining real-world objects as “complex objects”

When including complex objects, it is important to examine search behaviors between fully visible objects and visually incomplete objects at increasing object complexity (initially, from simple objects like rectangles to real-world objects that are slightly more complex like rectangular remote controllers). We are defining object complexity not as an object composed of an increasingly large number of components, but rather, an object embedded with categorical information, can have multiple versions easily recognized or recalled, and is associated with individual experiences with the object in real-world scenes (whether it is by physical interaction or visual observation).

### Possibilities of how complexity can affect search

#### Complexity may not support amodal completion

By increasing the stimuli’s complexity (e.g., using real-world objects such as Hershey bars and remote controllers), we can see if there are unique properties within an object that may deter pre-emptive grouping effects differently. For instance, searching for a remote controller or Hershey bar may be more difficult than search for a similarly shaped black bar. Additionally, this difficulty may occur when there is a white occluding bar but maybe not so when there is a gap. The white bar may interact with object surface features, and this interaction could interfere with surface completion (object segments’ color and texture) and edge completion (or “edge interpolation”) across the white occluder (Yin et al., 1997). If this interaction occurs, it could mean that search for objects with more complex surface features, such as multiple-colored geometric shapes (remote controller) and text (a Hershey bar) could be more disruptive than an object occluding a black bar. Therefore, if there is a gap in visible information, another object (such as a white bar as an occluder) does not need to be processed prior to the target, so pieces of real-world objects are grouped similarly after attention is deployed.

### Goals and hypothesis

#### Experiment 1

We tested whether there is a likelihood for a pop-out effect H_0_: $\delta \, = \text{ 0}$ (delta is the population search slope effect size) when completing orientation search for simple and complex objects that are completely visible and opaque (has no missing visual information). Wolfe et al.’s (2011) Experiment 1 showed that there was a pop-out effect (flat search slopes) when searching for a black bar’s orientation; and it is possible that search for a complex object’s orientation will yield evidence in favor of the null hypothesis (pop-out effect) over the alternative hypothesis (positive search slope). Wolfe et al.’s (2011) study demonstrated that white occluding bars are more disruptive to search than having a gap in the middle of the object. We test for evidence of the alternative hypothesis ${H}_{+}$: $\delta \text{ > 0}$ (difference in search slopes between searches with a white occluding bar and a gap) over the null (both similarly disruptive).

#### Experiment 2

Importantly, the intent of this study was to understand how a lack of visual information across targets affects both simple and complex objects. Though we tested for this effect in Experiment 1 (orientation search), feature search may be fundamentally different from identity search. For Experiment 2, we changed the task to an identity search with distractors that could be any other stimuli (e.g., Hershey bar target, with black bar and remote-control distractors) with any level of occlusion (stimuli with no visible disruptions, a gap in the middle of an object, or a white occluding bar over it). Because the task is no longer a feature search, we hypothesize that search for both simple and complex stimuli may yield evidence to be in favor of the alternative hypothesis (positive search slopes) over the null hypothesis (pop-out effect). In addition, we also test for evidence (for the alternative hypothesis over the null) in favor of differences between searches for occluded stimuli compared to stimuli containing a gap.

## Experiment 1

### Methods

#### Participants

Psychology undergraduate students (N = 34) were recruited from a mid-Atlantic University for course credit. All participants were naïve to the purpose of the studies.

### Materials

**Fig. 3:**
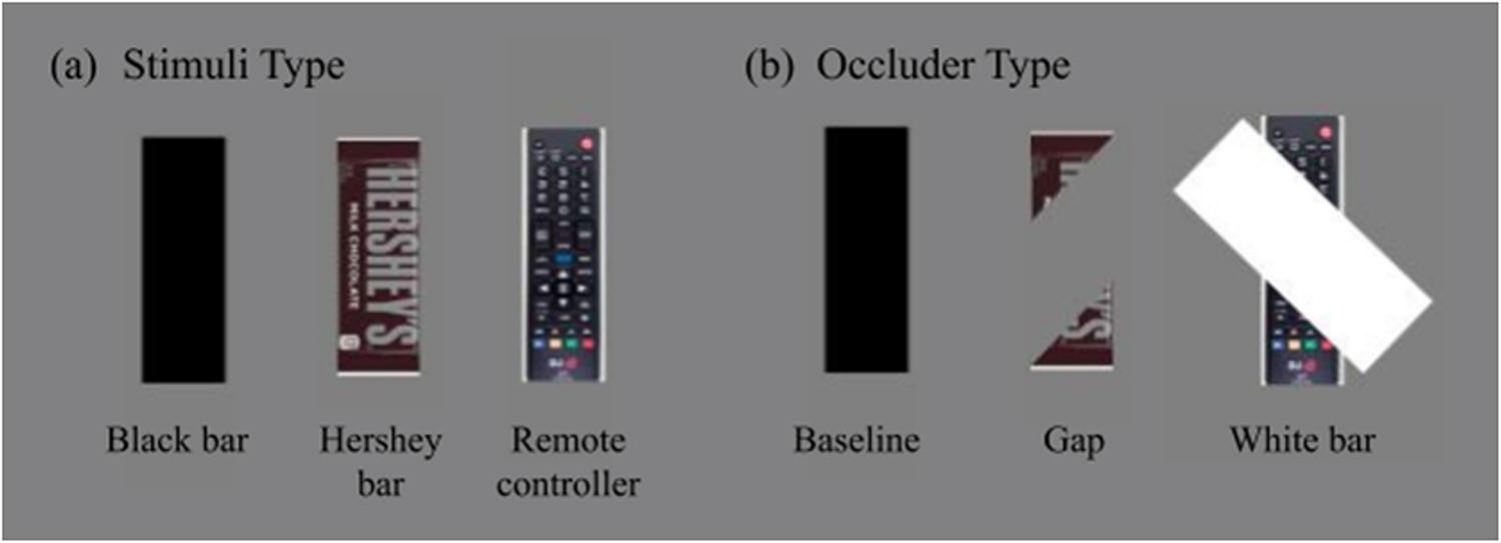
Stimuli and occluder types. For both Experiment 1 and Experiment 2 (**a**) three different stimuli were used as targets or distractors: Black bar, Hershey bar, and a remote. (**b**) Targets and distractors could have no partial occlusion (Baseline), a gray bar that matches the background color (Gap), or a white bar that partially occluded the stimuli

The experiment was created using Inquisit Software (Version 5), and participants used Inquisit Web (Version 5) to remotely access and complete the experiment in one sitting on their own computer. There were three different kinds of stimuli that were presented: a Black bar, a Hershey bar, and a rectangular remote controller; these stimuli were scaled to the same size and dimension. These stimuli were also either completely visible (Baseline), had a Gap in the middle of the stimuli (a gray bar, matching the gray-colored background, was diagonally superimposed over the image), or had a white bar that was diagonally placed over the image (see Fig. 3).

### Design

Experiment 1 was a 3 (Stimuli Type) × 3 (Occluder Type) × 2 (set size) within-subjects factorial design. The independent variables were the stimuli type (Black bar, Hershey bar, and remote controller) that were displayed, the Occluder type (Baseline, Gap, and White), and the number of stimuli presented on the screen (six or 12). The experiment was completed in a single session. Trials were presented in random order. The target was present for 50% of the experimental trials. There was an initial block of 24 practice trials with scenes that had a visual set size of 4 or 16; there were 720 experimental trials, with scenes that had a visual set size of 6 or 12, yielding a total of 745 experimental trials. The main task for this experiment was to search for the vertical target and to decide whether the vertical target was present or absent. Critically, distractors were horizontally orientated versions of the target with the same occluder type. This setup ensures that the participant is forced to look for the target’s orientation.

### Procedure

Before each session, participants were instructed to download Inquisit 5 Web (2016) and to complete the experiment in one sitting on their computer desktop or laptop. Visual instruction was given to each of the participants. They were instructed to stare at the initial and interstimulus fixation cross “+” before pressing the spacebar on their keyboard to begin the next trial. Before each experiment, a block of practice trials gave visual feedback based on the participant’s response. If a correct choice was made, “correct!” would appear and if the target was present, a green circle surrounded the target. If a wrong choice was made, “incorrect!” would appear and if the target was present, a red circle surrounded the target. If a wrong keypress was made, the program would not register that response, and if the trial time out, “No response detected” would be displayed in red. There were visual yellow texts with words, a “Yes” faced away from the center, towards the bottom left side of the screen, and a “No” faced away from the center, towards the bottom right of the screen to remind the participants which hand corresponded to which answer. After the block of practice trials ended, participants were reminded that the practice session had ended, and that the next experiment trials would display no feedback.

**Fig. 4:**
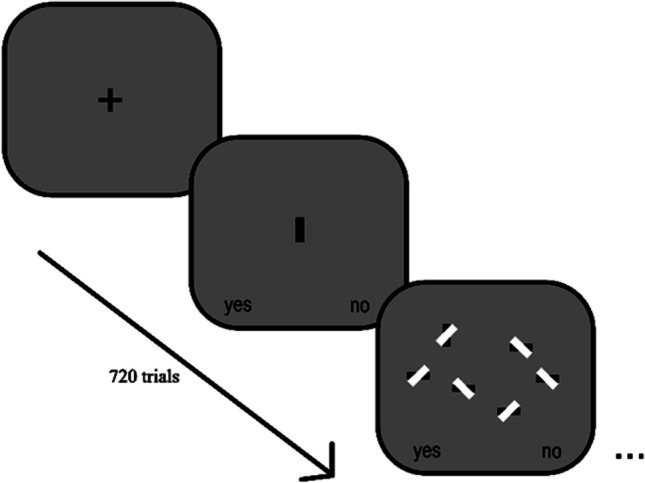
Experiment 1 trial progression. The task required participants to view the target before searching for the target within the scene

Before each experimental trial, the visual representation of the target participants had to search for was displayed (see Fig. 4). For instance, if the current trial was a black bar, a centered black bar was drawn where the interstimulus ‘+’ was displayed. Once the trial started, participants had 5 s to make a button press to decide whether a vertically aligned target was present or not present in a display with a visual set size per trial were either six or 12 items – the keyboard key “E” for choosing “Yes” and the keyboard key “I” for choosing “No.”

### Analysis

The dependent variable for the analysis is the *search slope* (Atkinson et al., 1969), which was computed by calculating the difference between averaged mean reaction time for the two visual set sizes (4 and 16) divided by the difference between the number of items present. Only reaction times for target-present trials were analyzed. Data were examined with JASP (2023) for outliers by looking at kurtosis, boxplots, and Q-Q plots for each of the conditions. Conditions Hershey-None (*k=1.66*), Hershey-Gap (*k=1.59*), and Remote-Gap (*k=2.61*) were leptokurtic with a kurtosis value greater than 1. Boxplots showed that the conditions Hershey-None, Remote-White, and Hershey-Gap had three outliers, and conditions Hershey-White bar and Remote-Gap had two outliers; one outlier from the Hershey-None condition was excluded because it was above six median absolute deviations around the median (Licata et al., 2013). Prior to observing the data, we assumed that the $\delta$ was distributed as a Cauchy distribution with scale of *r = 0.707* and prior odds of 1:1 for H_1_ and H_2._

### Results

We wanted to see if complex objects demonstrate a pop-out effect when performing a feature (orientation) search. If so, then search slopes will be close to zero, across increasing visual set sizes (6 to 12). Since the past experiment demonstrated flat search slopes when searching for a black bar’s orientation (Wolfe et al., 2011, Experiment 1), we checked to see if there would be evidence in favor of the null over the alternative hypothesis (${H}_{+0}$ ) for not just the simple object but also for complex objects. One-tailed one-sample t-tests (Faulkenberry & Wagenmakers, 2020; van Doorn et al., 2021) were conducted against the population mean of 0 in JASP (JASP Team, 2023) using the Bayes factor (${BF}_{+0}$). In testing for evidence of the likelihood of the null over the alternative hypothesis, we found that it was a little over six times more likely to result in the null over the alternative when searching for the orientation of a black bar ${BF}_{0+}$ *= 6.394* and remote controller ${BF}_{0+}$ *= 6.268*, and it was a little over three times more likely when searching for a Hershey bar ${BF}_{0+}$*= 3.197* (see Table 1).

**Table 1:** Bayesian t-tests conducted for orientation search against search slope of zero

Occluder type	Stimuli	Hypothesis	Bayes factor	$\delta$	$\delta$ 95% CI	Posterior model probability	Evidence
	Black	${H}_{0}$	(${BF}_{+0}$= 0.156) ${BF}_{0+}$= 6.394	0.098	[0.004, 0.343]	0.904	Moderate for ${H}_{0}$
None	Hershey	${H}_{0}$	(${BF}_{+0}$= 0.313) ${BF}_{0+}$= 3.197	0.154	[0.009, 0.445]	0.762	Moderate for ${H}_{0}$
	Remote	${H}_{0}$	(${BF}_{+0}$= 0.160) ${BF}_{0+}$= 6.268	0.099	[0.004, 0.346]	0.862	Moderate for ${H}_{0}$
	Black	${H}_{+}$	${BF}_{+0}$= 9.203 x 10^6^	1.324	[0.855, 1.808]	1.000	Extreme
Gap	Hershey	${H}_{+}$	${BF}_{+0}$= 20.939	0.500	[0.157, 0.857]	0.954	Strong
	Remote	${H}_{+}$	${BF}_{+0}$= 8.805	0.437	[0.109, 0.786]	0.898	Moderate
	Black	${H}_{+}$	${BF}_{+0}$= 1.610 x 10^5^	1.068	[0.641, 1.507]	1.000	Extreme
White Occluder	Hershey	${H}_{+}$	${BF}_{+0}$= 3.042 x 10^3^	0.824	[0.432, 1.224]	1.000	Extreme
	Remote	${H}_{+}$	${BF}_{+0}$= 1.810 x 10^4^	0.933	[0.526, 1.351]	1.000	Extreme

There are only pop-out effects (flat search slopes), for both simple and complex objects, when objects have no missing visual information. Strength of evidence from Jeffreys (1961) as cited by Faulkenberry & Wagenmakers (2020). The symbol $\delta$ denotes the population effect size. The posterior model with the probability for the preferred model assumes prior odds of 1:1

Additionally, we wanted to see if there is evidence in the likelihood of the alternative hypothesis over the null in searches for simple and complex objects containing missing visual information (whether there is a lack in information when there is a gap between object group components or if there is a white occluder bar through the object center). There is strong evidence for the alternative against the null hypothesis when performing an orientation search for objects that have missing visual information: extreme evidence for objects partially occluded by a white bar, moderate to extreme evidence for objects partially occluded by a gap, and moderate evidence for objects with no visual disruptions (Table 1).

**Fig. 5:**
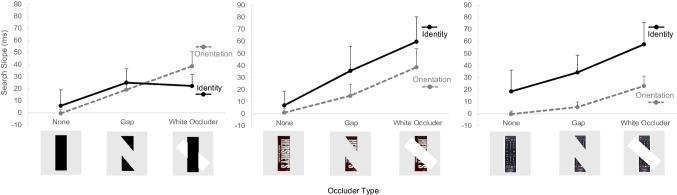
Orientation and identity search slope means across occluder types. Mean search slopes for each condition for Experiment 1 (orientation search) and Experiment 2 (identity search). Error bars are 95% credible intervals (only the upper limit is displayed for better clarity)

In addition to addressing our hypotheses, we wanted to see if there was evidence for the white occluder disrupting search more than a gap when searching for the orientation. White occluding bars were more disruptive to search than gaps of visual information in Wolfe et al.’s (2011) Experiment 1, so we hypothesized that there would be this difference between the two occluder types. Bayesian paired-samples t-tests were conducted post hoc, and there was extreme evidence for the high likelihood that white occluding bars are more disruptive than a gap of information (see Fig. 5) when searching for an object’s orientation (Table 2).

**Table 2:** Post hoc Bayesian analyses between occluder types and complex objects (orientation)

Comparison	Hypothesis	Bayes factor	δ	δ 95% CI	Posterior model probability	Evidence
Gap vs. White Occluder	${H}_{-}$: δ < 0 One-tailed alternative	${BF}_{-0}$= 2.012 x 10^4^	-0.495	[-0.701, -0.290]	1.000	Extreme for *H*_1_
Hershey bar vs. Remote controller	*H*_0_ Equivalent	*BF*_01_ = 0.548 *BF*_10_ = 1.825	0.234	[0.040, 0.428]	0.646	Anecdotal for *H*_1_

Search for orientation of a target with a white occluding bar is much more likely to be disruptive to a gap, and there is low to anecdotal evidence for a difference between searching for the orientation of different complex objects

Also, two visually similar complex objects were used as targets (Hershey bar and remote controller). Since we chose visually similar stimuli as a sample of different kinds of real-world objects, we did not expect there to be differences in search slopes between the two. A Bayesian paired-samples t-test was conducted between the two stimulus types. Evidence for a search difference between the two complex stimuli was only at the level of anecdotal evidence (*BF*_*10*_* =* 1.825).

## Experiment 2

### Methods

**Fig. 6:**
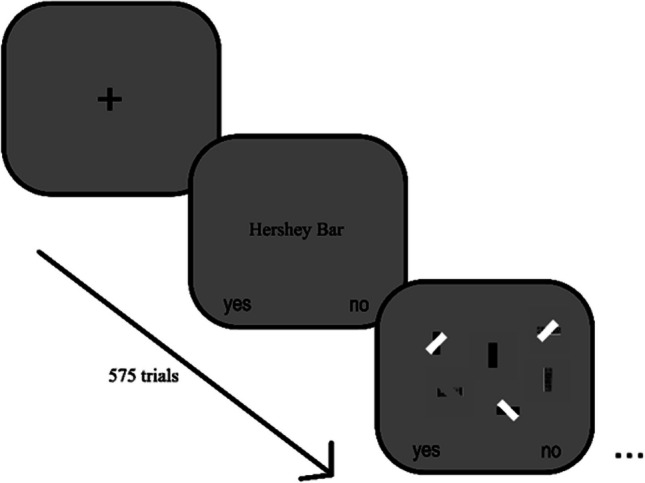
Experiment 2 trial progression. The task required participants to view the word name of the target before searching for the target within the scene

For Experiment 2, we changed the task from an orientation search to a target identity search (see Fig. 6). This task is potentially more difficult than the orientation task in Experiment 1 because a single feature, such as orientation, cannot be used to identify the target; instead, the entire object must be attended to identify whether the object is the target or a distractor. In Experiment 1, while it might be possible to identify a complex bar’s orientation by the orientation of its internal details, attending to the object’s internal details is critical for correct target identification for Experiment 2 (distractors are other non-target stimuli).

#### Participants

Psychology undergraduate students (N = 34) were recruited from a mid-Atlantic University for course credit, and N = 31 students’ data was kept; two students chose not to participate, and one student chose to end the experiment early. All participants were naïve to the purpose of the studies.

#### Materials and design

The same stimuli used in Experiment 1 was used in Experiment 2. Like Experiment 1, Experiment 2 was a 3 (Stimuli Type) × 3 (Occluder Type) × 2 (set size) within-subjects factorial design. Participants completed the experiment in one session. Trials were presented in random order, and the target was present for 50% of the experimental trials. We expected that the task would be more difficult and would take a longer time, so the total number of trials was decreased to minimize the possibility of fatigue affecting the effect of our independent variables. There was an initial block of nine practice trials with scenes that had a visual set size of 4 or 16, and there were 575 experimental trials with scenes that had a visual set size of 6 or 12, yielding a total of 584 experimental trials. Distractors consisted of two stimuli that were not the target, with any occlusion type. For example, if the target was a Hershey Bar, the distractor stimuli were Black Bars and Remotes that had no occlusion, had a gap, or were partially occluded by a diagonal white bar.

#### Procedure

Before each experimental trial, the words “Hershey Bar,” “Remote Controller,” or “Black Bar” were shown before the experimental trial (see Fig. 5). For instance, if the current trial was Hershey Bar, the word “Hershey Bar” was displayed in the middle of the screen. As with Experiment 1, participants had 5 seconds to make a button press to decide whether a vertically aligned target was present or not present in a display with a visual set size per trial of either six or 12 items. The keyboard key “E” for choosing “Yes” and the keyboard key “I” for choosing “No.” As with Experiment 1, participants only received feedback during the practice trials.

#### Analysis

Data were examined in a similar way to Experiment 1. Outliers were examined for conditions with kurtosis values greater than 1: Hershey-None (*k* = 1.126), Hershey-Gap (*k* = 3.853), Hershey-White (*k* = 4.797), Remote-Gray (*k* = 1.404), Remote-White (*k* = 3.042). Of the indicated outliers in JASP (JASP Team, 2023), one measure in Hershey-None and two measures in Remote-Gap were not included in the analysis because they were above five and a half median absolute deviations around the median (Licata et al., 2013)

### Results

We tested for pop-out effects (flat search slopes across increasing visual set sizes) when searching for all conditions, expecting targets that were completely visible (have no gap nor white occluding bar) to be easy to find. Surprisingly, there was anecdotal evidence for it to be more likely that searching for the remote controller would yield positive search slopes, while the null was more likely under the alternative hypothesis in search for black bars and Hershey bars (see Table 3). Observed data showed that search for both simple and complex objects that had any partial visual information missing is many times more likely to be under the alternative than null hypothesis.

**Table 3:** Bayesian t-tests conducted for identity search against search slope of zero

Occluder type	Stimuli	Hypothesis $\delta$ > 0	Bayes factor	δ	δ 95% CI	Posterior model probability	Evidence
	Black	${H}_{0}$	${BF}_{0+}$= 2.226	0.190	[0.012, 0.504]	0.690	Anecdotal for ${H}_{0}$
None	Hershey	${H}_{0}$	${BF}_{0+}$= 1.509	0.228	[0.017, 0.560]	0.601	Anecdotal for ${H}_{0}$
	Remote	${H}_{0}$	${BF}_{+0}$= 2.689 ${BF}_{0+}$= 0.372	0.355	[0.055, 0.710]	0.729	Anecdotal for ${H}_{1}$
	Black	${H}_{+}$	${BF}_{+0}$= 461.65	0.745	[0.347, 1.153]	0.998	Extreme
Gap	Hershey	${H}_{+}$	${BF}_{+0}$= 56.11	0.596	[0.219, 0.983]	0.982	Very Strong
	Remote	${H}_{+}$	${BF}_{+0}$= 1.339 x 10^3^	0.852	[0.424, 1.293]	0.999	Extreme
	Black	${H}_{+}$	${BF}_{+0}$= 1.003 x 10^3^	0.798	[0.392, 1.214]	0.999	Extreme
White Occluder	Hershey	${H}_{+}$	${BF}_{+0}$= 2.159 x 10^4^	1.006	[0.568, 1.456]	1.000	Extreme
	Remote	${H}_{+}$	${BF}_{+0}$= 9.112 x 10^4^	1.104	[0.650, 1.572]	1.000	Extreme

Searches for a simple object (black bar) and a complex object (Hershey bar) are more likely to result in a pop-out search. Search for a remote controller and any object with some visual missing detail has a high likelihood to result in a non-efficient search (positive search slopes)

As in Experiment 1, we conducted Bayesian paired-samples t-tests between the Gap and White Occluder and between complex objects (see Table 4). We expected that searching for an object that only had a gap of visual information would not be as challenging as searching for an object that was occluded by a white bar, and it was 3.7 times more likely for this to be true under the alternative hypothesis over the null. In addition, observed data comparing search slopes between Hershey bar and remote controller stimulus were 7.4 times more likely to occur under the null (equivalent search slopes) than alternative hypothesis. This result shows that a white occluding bar is not more disruptive to search than a gap of information when conducting identity search, and there effectively was no difference in search efficiency for the two complex stimuli Table 5.

**Table 4:** Post hoc Bayesian analyses between occluder types and complex objects (ID)

Comparison	Hypothesis	Bayes factor	δ	δ 95% CI	Posterior model probability	Evidence
Gap vs. White Occluder	${H}_{-}$: δ<0 One-tailed alternative	${BF}_{-0}$= 3.660	-0.273	[-0.478, -0.069]	0.785	Moderate for H_1_
Hershey bar vs. Remote controller	H_0_ Equivalent	BF_01_ = 7.343	-0.058	[-0.260, 0.145]	0.880	Moderate for H_0_

Search for target identity that contained a white occluding bar is likely to be disruptive, and there is moderate evidence for the likelihood of no differences between searching different types of complex objects

**Table 5:** Experiments 1 and 2 search slope means and credible intervals

		Experiment 1		Experiment 2	
Occluder type	Stimuli	Mean	95% CI	Mean	95% CI
	Black	-0.360	[-3.672, 2.951]	5.895	[-7.406, 19.200]
None	Hershey	0.967	[-2.363, 4.298]	6.989	[-4.780, 18.760]
	Remote	-0.243	[-2.802, 2.317]	18.495	[0.784, 36.200]
	Black	19.230	[14.381, 24.080]	25.059	[13.530, 36.589]
Gap	Hershey	14.909	[5.246, 24.572]	35.602	[15.291, 55.914]
	Remote	5.603	[1.451, 9.755]	34.319	[20.004, 48.634]
	Black	38.760	[26.721, 50.799]	22.325	[12.714, 31.936]
White Occluder	Hershey	38.647	[23.211, 54.083]	59.866	[39.250, 80.481]
	Remote	23.076	[14.909, 31.243]	57.556	[39.441, 75.672]

Search slope means and 95% credible intervals for each condition

**Table 6:** Experiments 1 and 2 means and credible intervals for collapsed conditions

	Experiment 1		Experiment 2	
Collapsed conditions	Mean	95% CI	Mean	95% CI
Gap White Occluder	13.248 33.494	[9.355, 17.140] [26.516, 40.470]	31.602 46.582	[20.780, 39.850] [36.600, 56.560]
Hershey bar Remote controller	17.904 9.479	[11.159, 24.650] [5.846, 13.110]	34.447 36.844	[23.420, 45.480] [26.860, 46.820]

Search slope means and 95% credible intervals for missing visual information collapsed across the two complex object types and object types collapsed across two of the missing visual information types

## General discussion

To better understand how occlusion or missing information affects our ability to search for objects, past studies have used simplistic or geometric shapes to test how amodal completion affected search; and while amodal completion can help complete length comparison tasks, it is inefficient (Rensink & Enns, 1995, 1998) and slows search for some objects (Wolfe et al., 2011). Specifically, having some visual disruption (blank space or object on top of the target) slows search for orientation; and having an object occlude a target was more disruptive than having a gap in the middle of an object (Wolfe et al., 2011, Experiment 1). To see if these visual disruptions also affect complex objects in a similar manner, we had participants search for the target feature, orientation (Experiment 1), as well as the target identity (Experiment 2). Additionally, we also checked to see if searching for a complex object’s orientation resulted in a pop-out effect for complex objects, similar to the pop-out effect seen in the search for whole, simple objects (Wolfe et al., 2011). The two experiments resulted in four main findings Table 6.

### Difference between feature search and object identity search

Overall, we found that searching for an object defined by a single feature, whether the object is simple or complex, is more likely to result in a pop-out effect, with the target easily found and appearing distinctive from distractors; this pop-out effect is highly likely to disappear once objects have partial missing visual information. In contrast to feature search, when the task is to search for target identity, searching for complex objects is likely to yield a more difficult search (where the target does not seem to pop out amongst distractors). Additionally, when there is missing visual information across any type of object, whether simple or complex, search becomes much more effortful.

Our results show that not only does feature search with simple objects yield pop-out effects across increasing visual set sizes (as in Wolfe et al.’s (2011) study), but this pop-out effect also occurs in feature searches with complex objects. When completing an orientation search, the target object outline is one of the defining features and may be easily accessible due to the global-superiority effect (Wagemans et al., 2012). Since all stimuli in orientation search were rectangular, it seems that the object’s surface area (or internal patterns) does not drive differences in search, and amodal completion does not automatically occur. In addition, when complex objects were not occluded and did not contain a gap, the evidence was unclear: search for a Hershey bar was efficient, but search for a remote controller was inefficient. This search efficiency dropped likely because there are more similarities between distractors in this task (Wolfe et al., 1989), and for partially visible targets, the target shared more similar parts with distractors (Alexander & Zelinsky, 2012).

Another reason could be that it becomes more difficult to decipher object membership when distractors with visual disruptions are present, and identity search relies on distinguishing the internal object texture between the target and distractors. A different task, such as comparing partially occluded object’s segment lengths (Rensink & Enns, 1995, 1998), shows that it can be difficult to distinguish between segment lengths because rapid grouping occurs between these segments. For our experiment, if the trial prompts the participant to search for a Hershey bar, and this target has some visual missing information, the Hershey bar can be found by amodal completion (white occluder condition) or by finding and knowing that the two visual pieces of the Hershey bar are part of the same Hershey bar (gap or white occluder conditions). When there are multiple distractors (in this example, black bars and remote controllers) with different occluder levels, it may be more difficult to detect a partially visible target because it may be more effortful to identify the target object’s pieces separately from the distractor’s members.

### Differences between complex objects for feature search, not identity search

Post hoc analysis also reveals that variability amongst complex objects is not likely to affect identity search, but there is an anecdotal likelihood that different types of complex objects may affect search speed when searching for an object feature (such as orientation). This result is potentially meaningful because objects that have a more complex surface area may affect amodal completion of a whole object. In addition, moderate evidence indicates that any differences in complexity between the Hershey bar and remote controller did not drive differences in search speed, suggesting that amodal completion with objects that contain an internal surface area of some complexity could affect search, even when the task involves searching for the object’s global shape. This effect (driven by the complexity) is also apparent in identity search, and differences between real-world objects with similar levels of complexity (in reference to internal surface areas) may not drive search differences. Since our real-world objects only consisted of Hershey bars and remote controllers, this inference is limited. There may or may not be a weak likelihood in search slope differences between other real-world objects.

### White occluders are more disruptive than gap for feature search

In addition, we wanted to see if having an invisible gap between an object was likely to have a different effect on search to having a white visible occluder on top of an object (white occluders were more disruptive than gaps in Wolfe et al., 2011, Experiment 1). Comparing the gap and white occluder conditions, we found that there was a very strong likelihood that having a visible occluder is more disruptive to search than having a gap when searching for an object feature. This strong likelihood occurred because amodal completion for the target below a white occluder was less efficient, possibly due the perception of depth between the target and white occluding bar. A target object that has a gap and/or a white occluding bar both result in the same amount of visible information that is missing (or visible). The difference between the two scenes is that the object with the occluding bar could be perceived to be at a different depth plane to the white bar. A second explanation for this likelihood could be that we process objects that have no visible disruptions before objects that have some visual discontinuity. Since the target’s overall outline is irrelevant to orientation search, our attention could be driven to the white occluding bar prior to target; and since objects with gaps do not have competing visibly completed objects, search slopes are not as deficient as search slopes for objects with white occluding bars.

**Fig. 7:**
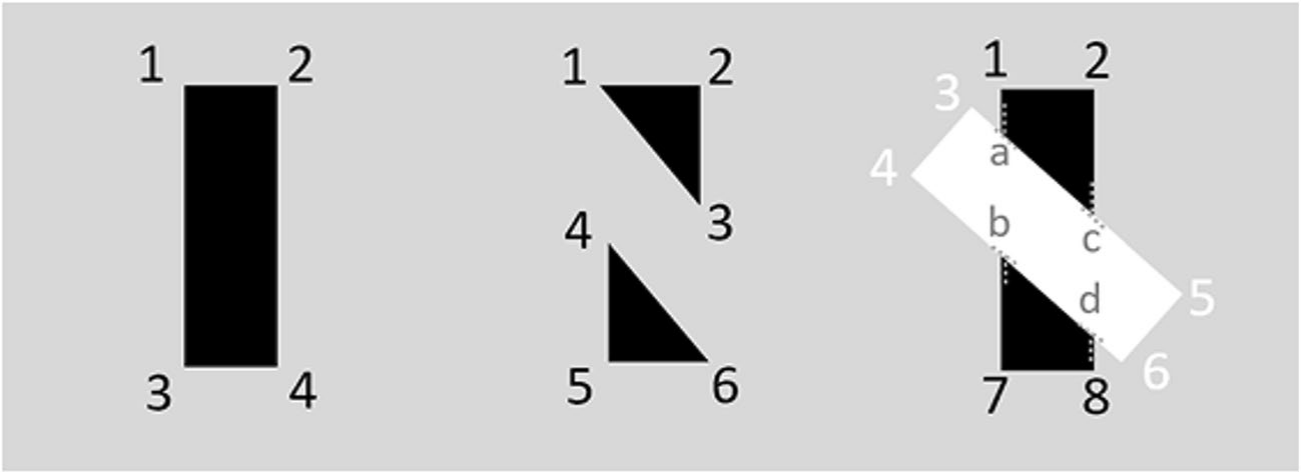
Gap and white occluder complexity. The baseline condition has four corners. The gap condition adds an additional two corners (total of six). The occluder condition contains a total of eight corners (four from the white occluder) and four T-junctions (**a–d**)

A third explanation is that white occluders are constructively more complex than gaps between objects (and both are more visually complex than the baseline condition with no visible missing areas). Gaps between the objects introduce two additional corners to an object, and white occluders introduce four additional corners from the white occluding bar for a total of eight corners (see Fig. 7). In addition to more corners, the white occluder also includes four T-junctions that increase its complexity. These constructive complexities could be a potential cofounding factor that makes search more challenging.

### Types of visible disruption affect identity search differently

Interestingly, there is a moderate likelihood that there will be a difference in effect on identity search between objects that have either of these two visible disruptions; additionally, in JASP (JASP Team, 2023), the sequential analysis plot (visual progression of data with different prior widths) shows that if the null hypothesis was true, there would be anecdotal to moderate evidence that looking for an object that has a gap of visible information or having a partially occluding object on top of it is *actually likely to have similar* search difficulties. If there are competing whole objects (white occluder bars) that are above some of the stimuli within the trial, these objects seem to potentially be processed prior to incomplete objects; therefore, searches were less efficient in the occluded condition compared to the gap condition. When the focus of the task is to distinguish a specific stimulus from others, a target with a visual disruption is harder to find than those that do not have one. Subsequent studies can investigate how these results hold for real-world object searches in various applied scenarios. Understanding how search behaviors can differ and change within different real-world scenarios (such as searching for crossing pedestrians, traffic signs, or vehicles in low lighting or turbulent weather) can help us to better understand visual search in complex environments.

## Limitations

To represent complex objects, we chose real-world objects that were similar in shape and overall color to a black bar, but with visible variation in the objects’ surface area (Hershey bar and remote controller). In theory, should we find evidence for the alternative hypothesis for those similar real-world objects, it is likely that this evidence would extend towards complex objects that have more visual variability compared to a black bar. The limitation to using complex objects similar in shape and size to the simple object is that these results might not extend to other real-world objects different in overall shape and color. Our results could be considered “conservative” because results that include evidence in favor of the null hypothesis for complex objects may or may not hold for other real-world objects with greater visual variability to a black bar. For example, there was moderate evidence for the null hypothesis between complex objects in identity search (Experiment 2). If we used a different real-world object (such as a plant), it is possible that there would still be evidence for the null hypothesis, but it is also possible that there could be anecdotal evidence for the alternative hypothesis. Though the complex stimuli used could be a possible limitation, it could be a strength because evidence for alternative hypotheses may likely hold for other complex objects with higher visual variability to simple objects. In addition, the use of similarly shaped complex objects outweighs additional analyses for better comparisons specific to our tasks (i.e., orientation search).

## Data Availability

Not applicable.

## Declarations

### Open practices statement

The datasets generated and/or analyzed during the current study are available from the corresponding author on reasonable request, and neither experiments were preregistered.
